# Effective Oral Indicators With Medical and Dental Collaboration in Open Heart Surgery: A Representative Survey

**DOI:** 10.7759/cureus.62392

**Published:** 2024-06-14

**Authors:** Kaori Ono, Shouji Hironaka, Akemi Utsumi, Asako Yamaguchi, Yumi Shibata, Luna Osakabe, Shuichiro Oka, Atsushi Aoki, Toru Kotani, Kyoko Shirakura, Satoko Yamaguchi, Mie Myers, Yasubumi Maruoka

**Affiliations:** 1 Department of Perioperative Medicine, Showa University School of Dentistry, Tokyo, JPN; 2 Department of Hygiene and Oral Health, Showa University School of Dentistry, Tokyo, JPN; 3 Department of Hospital Dentistry, Showa University Hospital, Tokyo, JPN; 4 Graduate School of Health Sciences, Showa University, Kanagawa, JPN; 5 Department of Dental Anesthesia, Showa University Hospital, Tokyo, JPN; 6 Department of Perioperative Medicine, Division of Anesthesiology, Showa University School of Dentistry, Tokyo, JPN; 7 Department of Surgery, Showa University School of Medicine, Tokyo, JPN; 8 Department of Intensive Care Medicine, Showa University School of Medicine, Tokyo, JPN; 9 Department of Oral and Maxillofacial Surgery, Totsuka Kyoritsu Daini Hospital, Kanagawa, JPN

**Keywords:** oral assessment, oral bacteria, hospitalized patients, perioperative oral management, postoperative infection, postoperative complications, surgery, bacteria, oral health, cardiovascular diseases

## Abstract

Purpose

Postoperative infections pose an important problem for patients with cardiac disease. Moreover, oral health status is associated with the risk of longer hospital stays. Therefore, the oral health status of patients was assessed before open-heart surgery. This study aimed to determine the relationship between oral health status and postoperative status.

Methods

The study included 25 patients who underwent open-heart surgery at our university hospital in 2020. Upon admission, dentists conducted an oral examination and assessed the oral health status of the patients, also using the Japanese version of the Oral Health Assessment Tool (OHAT-J), Revised Oral Assessment Guide (ROAG), oral moisture level, oral bacteria, and other relevant factors. The study investigated the association with postoperative status.

Findings

Significant postoperative infections were found in patients aged ≥70 years, with an OHAT-J score of ≥5, OHAT-J lip score of ≥1, *Streptococcus* γ count of 1.0 × 10^6 or higher (CFU/mL), and increased *Streptococcus* γ before and after surgery. The duration of hospitalization correlated with the OHAT-J, OHAT-J gum and tissue, and ROAG scores. The duration of intensive care unit (ICU) stays correlated with the OHAT-J score.

Conclusions

The study demonstrates that OHAT-J scores are linked with predicting not just postoperative infection but also the length of hospitalization and ICU stay. As OHAT-J scores do not necessitate specialized dental instruments, they are straightforward and beneficial for healthcare professionals outside of dentistry.

## Introduction

Postoperative infections, such as postoperative fever, wound infections, and lower respiratory tract infections, are common in cardiovascular surgery because of their highly invasive nature [1-4]. These complications, including postoperative infections, can decrease the postoperative quality of life of the patients [5,6]. They can also increase hospital resource consumption because of costs and length of stay [7]. Oral bacteria, such as Streptococcus mutans, are often responsible for postoperative infections after cardiovascular open-heart surgery procedures such as heart valve replacement, aortic aneurysmectomy, and prosthetic valve endometriosis [1,8].

Improving perioperative oral health management (POM) can promote the postoperative quality of life of the patients [4,9-13]. In our previous study, we found that patients with a family dentist had a shorter length of stay than those without a family dentist [6]. Therefore, POM was implemented in Japan in April 2012 to reduce postoperative complications, such as aspiration pneumonia. At Showa University Hospital, through medical and dental collaboration, oral care was provided by dentistry to patients undergoing cardiovascular surgery [6,14].

However, which oral conditions are associated with postoperative status in patients undergoing cardiovascular surgery remained unclear. Therefore, this study aimed to examine the oral health status of patients undergoing cardiovascular surgery to identify characteristics of those more likely to experience poor postoperative status and predict those who would benefit from intensive oral care.

## Materials and methods

Study design and participants

The experimental protocol employed in this study was approved by the Ethics Review Committee for Research in Human Subjects, Showa University Graduate School of Medicine (Approval no. 3033). The study protocol was performed in accordance with the Declaration of Helsinki.

This prospective observational study included all patients who underwent POM between March and September 2020 at the request of the Department of Cardiovascular Surgery to the Department of Dentistry of Showa University Hospital. Out of the 57 patients who provided written consent, 32 were excluded from the analysis for the following reasons: non-open chest surgeries (n=30), pre-surgery mortality (n=1), and lack of oral microbiota data (n=1).

**Figure 1:**
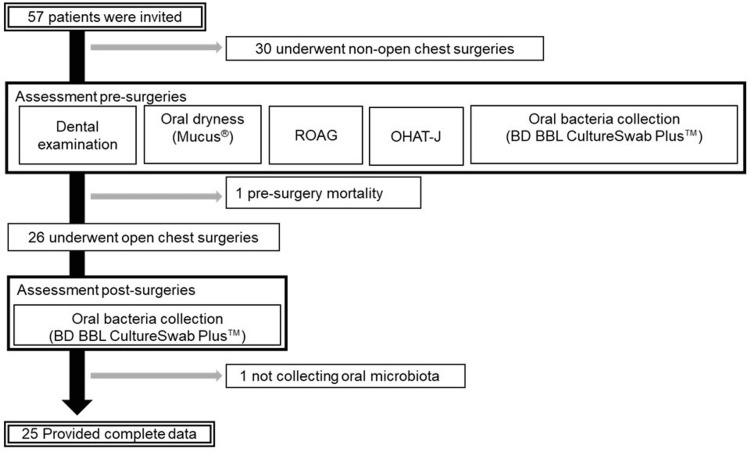
Flow diagram ROAG - Revised Oral Assessment Guide; OHAT-J - the Japanese version of the Oral Health Assessment Tool

Eventually, 25 participants, including 14 male and 11 female patients, were included in the study for further analysis (Figure 1).

Methods of oral health status assessment

The assessment items for the general and oral health status are shown in Figure 1. Before admission, patients' oral cavities were assessed when they visited the Department of Dental and Oral Surgery by six experienced dentists and one dental hygienist. Seven dental professionals were calibrated before the study. All patients received perioperative antibiotics and steroid therapy [15].

Patient information, including age, sex, primary diseases, surgery procedures, comorbidities, height, weight, body mass index, duration of hospitalization, and duration of intensive care unit (ICU) stay, was collected from the medical records of the Department of Cardiovascular Surgery. The primary diseases were classified with reference to the International Statistical Classification of Diseases and Related Health Problems, 11th Revision [16]. However, there might be duplications in both primary diseases and surgical procedures.

The following general oral findings were assessed: tooth number, periodontal pocket measurements, bleeding on probing, and whether or not the patient wore dentures. The Japanese version of the Oral Health Assessment Tool (OHAT-J) and Revised Oral Assessment Guide (ROAG) were used for the oral health assessment [17-21].

The oral moisture analyzer Mucus® (Life, Saitama, Japan) was used to evaluate oral dryness [22]. The presence or absence of postoperative infection was determined by referencing the medical records of the Department of Cardiovascular Surgery.

Sampling and oral bacteria analysis

Oral bacterial samples were collected from the patients twice: once before surgery and once after surgery. Postoperative sampling was conducted within 48 hours of the surgery, and all patients had been extubated. Oral bacteria samples were obtained from the tongue's dorsal surface using BD BBL CultureSwab Plus™ (Becton, Dickinson and Company, Franklin Lakes, New Jersey). Bio Medical Laboratories, Inc. (BML, Tokyo, Japan) analyzed the samples [14]. The material was cultured in different media at specific conditions, the target bacteria were isolated after 17-20 h at 37°C and cultured again, and their properties were examined using various media.

Statistical analysis

All quantitative variables are presented as medians and interquartile ranges. The oral bacterial count of 10 × 10^3 or less was considered "no detection." The median count was 10 × 10^3 or less, so this value was defined as no detection. The relationship between postoperative infection and oral health status, including OHAT-J, ROAG, oral bacteria, and other factors, was analyzed using the chi-square test. The χ2, ϕ coefficient, and p-values were estimated. The correlations between oral health status and variables such as the duration of hospitalization, duration of ICU stay, and postoperative infection were examined using Spearman's correlation coefficients.

All analyses were conducted using SPSS statistics version 26.0 (IBM Inc., Armonk, New York). In all analyses, p-values of <0.05 indicated statistical significance.

## Results

Patient characteristics

Table 1 shows the demographic characteristics of the patients. There were 14 male and 11 female patients, with a median age of 70.0 (range 62.0-78.0) years.

**Table 1:** The demographic characteristics of patients

Demographic Characteristics	Number of patients/median (interquartile range)	Range
Age in years	70 (62.0 - 78.0)	48 - 85
Sex, n (%)		
Male	14 (56.0%)	
Female	11 (44.0%)	
Height (cm)	161.3 (153.00 - 171.68)	143.5 - 176.3
Body weight (kg)	54.8 (49.70 - 70.40)	39.6 - 94.3
Body mass index	23.15 (20.14 - 24.59)	17.88 - 31.51

Table 2 shows the clinical characteristics of the patients. The most common diagnosis was aortic valve stenosis (n=11), and the most common surgical procedure was an aortic valve replacement (n=13). The median duration of ICU stay was 3.0 (range 3.0-3.0) days, and the median duration of hospitalization was 33 (range 21.0-49.0) days. In total, eight postoperative infections occurred, comprising one case of infective endocarditis and seven cases of urinary tract infections.

**Table 2:** The clinical characteristics of the patients Duplicate counts were made for primary diseases, surgery procedures, and co-morbid diseases. For convenience, no detection is indicated by 1.0x10^3. ROAG - Revised Oral Assessment Guide; OHAT-J - the Japanese version of the Oral Health Assessment Tool; ICU - intensive care unit; CFU - colony forming unit

Clinical characteristics	Number of patients/median (interquartile range)	Range
Primary diseases, n		
Aortic valve stenosis	11	
Aortic valve insufficiency	6	
Congestive heart failure	4	
Mitral valve insufficiency	4	
Mitral valve stenosis	4	
Surgery procedures, n		
Aortic valve replacement	13	
Mitral valve replacement	9	
Tricuspid valve repair	6	
Artificial vessel replacement	3	
Mitral valve replacement	3	
Co-morbid diseases, n		
Digestive disorders	10	
Metabolic disorders	9	
Orthopedic diseases	4	
Gynecological diseases	4	
Respiratory disease	3	
Neuromuscular disease	3	
Oral health states		
Number of present tooth, n	26 (23.00 - 28.00)	0 - 30
Percentage of periodontal pockets greater than 4mm (%)	11.5 (0.00 - 21.70)	0 - 100
Bleeding on probing (%)	15.4 (6.90 - 28.60)	0 - 100
Dentures, n		
Two dentures	3	
One denture	1	
No dentures	21	
ROAG	11 (9 - 11)	8 - 15
Oral moisture degree	28.1 (26.30 - 30.10)	2.9 - 32.3
OHAT-J		
Total score	4 (3 - 5)	1 - 9
Lip	0 (0 - 0)	0 - 1
Tongue	1 (0 - 1)	0 - 1
Gum and tissues	0 (0 - 2)	0 - 2
Saliva	0 (0 - 0)	0 - 1
Number of present tooth	1 (0 - 1)	0 - 2
Dentures	0 (0 - 0)	0 - 1
Oral cleanliness	2 (1 - 2)	0 - 2
Dental pain	0 (0 - 0)	0 - 0
The duration of postoperative events		
The duration of intubation period	1 (1.00 - 1.00)	0 - 2
The duration of ICU stay	3 (3.00 - 3.00)	2 - 13
The duration of Respiratory management	4 (3.00 – 5.00)	2 - 6
Days to start of oral feeding	1 (1.00 – 2.00)	0 - 3
The duration of hospitalization (days)	33 (21.00 – 49.00)	12 - 82
Oral bacteria		
*Streptococcus *α (CFU)	4.3x10^6 (1.1x10^6 - 1.4x10^7)	1.4x10^5 - 7.3x10^7
*Streptococcus *γ (CFU)	3.4x10^7 (2.3x10^6 - 4.9x10^7)	1.0x10^3 - 1.2x10^8
Anaerobic bacteria (*Prevotella *sp.) (CFU)	1.0x10^3 (1.0x10^3 - 1.3x10^5)	1.0x10^3 - 4.3x10^6

Factors related to postoperative infection

Table 3 compares the patients with postoperative infection (n=8) with those without postoperative infection (n=17). No significant difference was found between the groups in the type of drugs received.

**Table 3:** Factors related to postoperative infection ROAG - Revised Oral Assessment Guide; OHAT-J - the Japanese version of the Oral Health Assessment; CFU - colony forming unit

Variable		No postoperative infection (N = 17)	Postoperative infection (N = 8)	Χ2	p-value	Φ
Age (years)	<70	5	6	4.588	0.032*	-0.157
	≥70	12	2			
Sex	Male	10	4	0.172	0.678	0.083
	Female	7	4			
Body mass index	≥21.5	10	6	0.618	0.432	0.157
	<21.5	9	2			
Number of present tooth, n	≥27	9	3	0.520	0.471	0.144
	≤26	8	5			
Periodontal pockets greater than 4mm	(-)	13	6	0.164	0.686	0.081
	(+)	4	2			
Bleeding on probing (%)	<25	11	4	0.490	0.484	0.140
	≥25	6	4			
Denture use	(-)	13	8	2.241	0.137	0.298
	(+)	4	0			
ROAG	≤8	2	0	1.023	0.186	-0.265
	≥9	15	8			
Oral moisture degree	≥27	12	5	0.164	0.134	-0.299
	<27	5	3			
OHAT-J						
Total score	≤4	12	2	4.588	0.032*	0.428
	≥5	5	6			
Lip	0	17	6	4.620	0.032*	0.430
	≥1	0	2			
Tongue	0	7	3	0.031	0.861	0.035
	≥1	10	5			
Gum and tissues	0	11	3	1.634	0.201	0.256
	≥1	6	5			
Saliva	0	15	6	0.790	0.400	0.168
	≥1	2	2			
Number of present tooth	0	7	3	0.310	0.861	0.035
	≥1	10	5			
Dentures	0	17	7	2.214	0.137	0.298
	≥1	0	1			
Oral cleanliness	≤1	8	1	2.820	0.093	0.336
	≥2	9	7			
Dental pain	0	18	8			
	≥1	0	0			
Oral bacteria						
Streptococcus α	≥1.0x10^6	3	3	1.176	0.278	-0.217
(CFU)	<1.0x10^6	14	5			
Streptococcus γ	≥1.0x10^6	0	4	6.618	0.010*	0.020
(CFU)	<1.0x10^6	17	4			
Anaerobic bacteria	("no detection")	12	5	0.164	0.686	0.081
(*Prevotella sp*.)	(+)	5	3			
Change in *Streptococcus α *count	(+)	13	5	0.527	0.468	0.145
	(0~-)	4	3			
Change in *Streptococcus γ* count	(+)	16	5	4.046	0.044*	0.402
	(0~-)	1	3			
Change in *Prevotella sp*. count	(+)	14	5	1.176	0.278	0.217
	(0~-)	3	3			

Significant postoperative infections were found in patients aged ≥70 years (p=0.032), with OHAT-J scores of ≥5 (p=0.032), OHAT-J Lip scores of ≥1 (p=0.032), Streptococcus γ count of 1.0 × 10^6 or higher (CFU/mL) (p=0.010), and an increase in Streptococcus γ before and after surgery (p=0.044). No other significant differences were found between the groups.

Correlations between the factors and the durations of hospitalization and ICU stay

Table 4 displays correlations between the factors and the duration of hospitalization and ICU stay. The duration of hospitalization correlated with the OHAT-J (p=0.033), OHAT-J score, OHAT-J gum and tissue (p=0.005), and ROAG (p=0.032) scores. The duration of ICU stay correlated with the OHAT-J score (p=0.032). No other significant correlations were found.

**Table 4:** Correlations between the factors and the duration of hospitalization and ICU stay ICU - intensive care unit; OHAT-J - the Japanese version of the Oral Health Assessment Tool; ROAG - Revised Oral Assessment Guide

Variable	The duration of hospitalization		The duration of ICU stay	
	Correlation coefficient	p-value	Correlation coefficient	p-value
Age (years)	0.216	0.126	0.292	0.055
Body mass index	0.064	0.369	-0.014	0.47
Number of present tooth	-0.048	0.401	0.1	0.297
Number of teeth with periodontal pockets greater than 4mm	0.026	0.446	0.245	0.092
Bleeding on probing (%)	0.075	0.348	0.143	0.222
Oral moisture degree	-0.156	0.205	-0.077	0.341
OHAT-J	0.34	0.033*	0.312	0.044*
Lip	0.043	0.411	0.118	0.264
Tongue	0.264	0.08	-0.099	0.299
Gum and tissues	0.468	0.005*	0.204	0.135
Saliva	0.056	0.384	0.066	0.361
Number of present tooth	-0.154	0.208	-0.05	0.395
Dentures	0.053	0.391	-0.095	0.306
Oral cleanliness	0.145	0.223	0.256	0.082
ROAG	0.418	0.011*	0.234	0.103
*Streptococcus α* (CFU)	-0.193	0.356	0.042	0.843
*Streptococcus γ *(CFU)	-0.226	0.278	0.113	0.592
Anaerobic bacteria	-0.046	0.827	-0.043	0.837
(*Prevotella sp*.) (CFU)				

## Discussion

Association between OHAT-J scores and postoperative infection and the duration of hospitalization and ICU stay

This study found that postoperative infections are significantly associated with age, OHAT-J scores, and OHAT-J lip scores. Thus, assessing age, OHAT-J scores and OHAT-J lip scores can help predict postoperative infection. OHAT-J scores were also significantly correlated with the length of hospitalization and ICU duration. Therefore, OHAT-J scores may be useful in predicting not only postoperative infection but also length of hospitalization and ICU duration.

Initially, we hypothesized that reduced saliva production and progressive oral frailty might lead to dry lips. However, our findings revealed a significant difference in OHAT-J lip scores but not in oral moisture levels among patients with postoperative infections. The OHAT-J lip scores might be easier to assess among the categories of the OHAT-J scores because the lips are located outside the oral cavity.

Then, we hypothesized that patients with periodontal disease would be more prone to postoperative infections and prolonged hospitalization. Therefore, we measured various periodontal indicators. However, specialized measures of periodontal disease, such as the number of teeth with pockets >4 mm and bleeding on probing, showed no significant correlation. On the contrary, OHAT-J scores and OHAT-J gum and tissue scores correlated with the length of hospitalization, and OHAT-J scores correlated with the length of ICU stay. OHAT-J gum and tissue scores were simple and useful for non-dental health care professionals because they do not require special dental instruments.

In addition, the length of hospitalization correlated with ROAG. Because ROAG specifically assesses oral hygiene in patients with cancer, it might be too strong as an indicator for patients undergoing open-heart surgery [20].

POM has become common in the dental field since it was implemented in Japan approximately 10 years ago [12,13]. The OHAT-J scores facilitate the evaluation of the oral health state by non-dental healthcare professionals [17,18,23]. Therefore, OHAT-J scores may initially be more useful for non-dental health care professionals than oral moisture levels and specialized measures for periodontal diseases as a predictive indicator of postoperative infection and long-term hospitalization.

Oral bacteriological analysis

In this study, oral bacteria were evaluated; however, only normal bacteria flora was detected, and specialized or harmful bacteria were not found. In highly invasive treatments such as cardiac surgeries, indigenous oral bacteria commonly cause opportunistic infections because of perioperative antibiotic and steroid therapy [3,8,15]. This study showed that individuals with high preoperative levels of *Streptococcus γ* and those with an increase in *Streptococcus γ* before and after surgery were significantly different compared with those without postoperative infections. When overall health deteriorates, oral bacteria like *Streptococcus *may increase [24,25]. For example, Oral *Streptococcus *are pathogens associated with various systemic diseases, including infective endocarditis, purulent infections, and bacteremia [24,25]. *Streptococcus γ* is a common oral bacterium; however, it can lead to oral infections because of its solubility [26]. Thus, maintaining good oral health is important even before surgery.

More effective medical and dental collaboration on open-chest surgery

The guideline on the management of valvular heart disease, developed by the Japanese Cardiology Society and other organizations in 2020, emphasizes the significance of assessing frailty in older individuals to determine the appropriate surgical technique [15]. Previous studies have indicated a correlation between frailty and cardiac surgical outcomes, particularly among older patients [27,28]. In this study, infections developed in some patients postoperatively despite being assessed preoperatively as having a low risk of frailty and a low risk of surgery. Oral frailty is a preliminary stage of frailty [12]. Thus, oral frailty must be evaluated as one of the risk factors for surgery in older people because predicting the condition of the patient after the is very important.

Limitations

This study has some limitations. First, various aspects of oral function, such as tongue pressure, occlusal force, lip-closure strength, and oral diadochokinesis, were not assessed. Thus, additional experiments for future studies are planned to address this gap. Second, the study has a small sample. Finally, the backgrounds of the participants were not considered because of the limited number of participants.

The time period of this study coincided with the COVID-19 epidemic, which affected the number of hospitalized patients. We are planning a multi-institutional study that will include our affiliated hospitals because exploring different populations and settings could provide a broader understanding of the topic. Future research could investigate additional variables that may impact the outcomes, building on the insights gained from this study.

## Conclusions

The study shows that OHAT-J scores are associated with predicting not only postoperative infection but also length of hospitalization and ICU stay. Because OHAT-J scores do not require special dental instruments, they are simple and useful for non-dental healthcare professionals.

Therefore, the OHAT-J scores may be a predictive indicator in achieving more effective medical and dental collaboration in open-chest surgery. Furthermore, we would argue that smoother medical and dental collaboration is more beneficial to the patients.
